# Risk Factors for Mortality among Adult HIV/AIDS Patients Following Antiretroviral Therapy in Southwestern Ethiopia: An Assessment through Survival Models

**DOI:** 10.3390/ijerph14030296

**Published:** 2017-03-12

**Authors:** Dinberu Seyoum, Jean-Marie Degryse, Yehenew Getachew Kifle, Ayele Taye, Mulualem Tadesse, Belay Birlie, Akalu Banbeta, Angel Rosas-Aguirre, Luc Duchateau, Niko Speybroeck

**Affiliations:** 1 Institute of Health and Society (IRSS), Université catholique de Louvain, Brussels B-1082, Belgium; Jean-Marie.Degryse@uclouvain.be (J.-M.D.); angelrosasa@gmail.com (A.R.-A.); niko.speybroeck@uclouvain.be (N.S.); 2 Department of Statistics, Natural Science College, Jimma University, Jimma, PO Box 378, Ethiopia; belaya.birlie@gmail.com (B.B.); akalubanbeta.stat@gmail.com (A.B.); 3 Department Public Health and Primary Care, KU Leuven, Leuven B-3000, Belgium; 4 Department of Statistics and Operations Research, University of Limpopo, Sovenga, 0727, South Africa; yehenew.getachew@yahoo.com; 5 School of Mathematical and Statistical Science, Hawassa University, P.O. Box 05, Ethiopia; ayele_taye@yahoo.com; 6 Department of Medical Laboratory Sciences and Pathology, College of Health Sciences, Jimma University, Jimma, P.O. Box 378, Ethiopia; mulualemt.tadesse@gmail.com; 7 Institute of Tropical Medicine “Alexander von Humboldt”, Universidad Peruana Cayetano Heredia, Lima 15000, Peru; 8 Department of Comparative Physiology and Biometrics, Ghent University, Ghent B-9000, Belgium; luc.duchateau@ugent.be

**Keywords:** antiretroviral therapy (ART), HIV/AIDS, parametric survival model, Ethiopia

## Abstract

*Introduction:* Efforts have been made to reduce HIV/AIDS-related mortality by delivering antiretroviral therapy (ART) treatment. However, HIV patients in resource-poor settings are still dying, even if they are on ART treatment. This study aimed to explore the factors associated with HIV/AIDS-related mortality in Southwestern Ethiopia. *Method:* A non-concurrent retrospective cohort study which collected data from the clinical records of adult HIV/AIDS patients, who initiated ART treatment and were followed between January 2006 and December 2010, was conducted, to explore the factors associated with HIV/AIDS-related mortality at Jimma University Specialized Hospital (JUSH). Survival times (i.e., the time from the onset of ART treatment to the death or censoring) and different characteristics of patients were retrospectively examined. A best-fit model was chosen for the survival data, after the comparison between native semi-parametric Cox regression and parametric survival models (i.e., exponential, Weibull, and log-logistic). *Result:* A total of 456 HIV patients were included in the study, mostly females (312, 68.4%), with a median age of 30 years (inter-quartile range (IQR): 23–37 years). Estimated follow-up until December 2010 accounted for 1245 person-years at risk (PYAR) and resulted in 66 (14.5%) deaths and 390 censored individuals, representing a median survival time of 34.0 months ( IQR: 22.8–42.0 months). The overall mortality rate was 5.3/100 PYAR: 6.5/100 PYAR for males and 4.8/100 PYAR for females. The Weibull survival model was the best model for fitting the data (lowest AIC). The main factors associated with mortality were: baseline age (>35 years old, AHR = 3.8, 95% CI: 1.6–9.1), baseline weight (AHR = 0.93, 95% CI: 0.90–0.97), baseline WHO stage IV (AHR = 6.2, 95% CI: 2.2–14.2), and low adherence to ART treatment (AHR = 4.2, 95% CI: 2.5–7.1). *Conclusion:* An effective reduction in HIV/AIDS mortality could be achieved through timely ART treatment onset and maintaining high levels of treatment adherence.

## 1. Introduction

HIV/AIDS continues to be a major global public health issue and thus far, has claimed the lives of more than 34 million people worldwide. In 2014, approximately 1.2 (1.0–1.5) million people died from a HIV-related causes [1]. Sub-Saharan Africa was the most affected region, with 25.8 (24.0–28.7) million people living with HIV in 2014; the region accounts for nearly 70% of new HIV infections globally [1]. In Ethiopia, it has been estimated that approximately 45,200 (36,500–55,200) deaths were related to AIDS and that 793,700 (716,300–893,200) people were living with HIV in 2013 [1]. Effective treatment with antiretroviral (ARV) drugs can control the infection and the disease, and allow HIV-infected people to enjoy healthy and productive lives. Antiretroviral therapy (ART) reduces HIV replication and the infection of new cells, and it improves the immune system function. Therefore, ARV therapy positively influences the quality of life and the survival of seropositive HIV carriers [2].

Different clinical, demographic, socio-economic, and behavioural factors have been reported to be related to the survival of HIV-infected patients under ART [3,4]. In Sub-Saharan Africa, studies have reported an important correlation between mortality in HIV-patients under treatment and late diagnosis of HIV, the delay in ART initiation, an advanced World Health Organization (WHO) clinical stage, low CD4 counts, high viral loads, a low body weight, low haemoglobin levels, and poor socio-economic conditions [5,6,7,8]. Gender and migration have also been associated with the risk of dying in seropositive individuals in South Africa [4]. In Ethiopia, factors such as severe anemia, a history of co-infection with tuberculosis (TB), marital status, WHO stage, low CD4 counts, poor adherence to ART, substance use, and opportunistic infections, were also found to be important determinants of HIV/AIDS-related deaths [9,10,11,12].

Both semi-parametric and parametric survival models have been used to predict the effects of different potentially influential factors on the time until HIV/AIDS-related death (survival time) [3,9]. Although the Cox regression model has been widely used for this purpose due to its minimal requirements of assumptions for predicting the prognostic factors associated with survival [3,13], parametric models have been demonstrated to be more accurate in creating projections regarding the risk of mortality beyond the observed follow-up period [14,15]. In this paper, we report the results obtained using three of the most popular parametric survival models (i.e., exponential, Weibull, and log-logistic) and one semi-parametric (Cox proportional hazard model) model; these models analyzed non-concurrent retrospective cohort data from patients with HIV/AIDS, who initiated ART treatment between January 2006 and December 2010 at the Jimma University Specialized Hospital (JUSH) in Ethiopia, in order to identify the predictive factors of HIV/AIDS-related mortality.

## 2. Methods

### 2.1. Study Setting

Jimma University Specialized Hospital (JUSH) is one of the oldest public referral hospitals in Ethiopia, which was established in 1930 by Italian invaders to provide medical services to their soldiers. JUSH is located in Jimma City, 352 km southwest of Addis Ababa. According to the census conducted by the Central Statistics Agency of Ethiopia (CSA) in 2007, Jimma City had a total population of 120,960 inhabitants living in an area of approximately 50.5 km^2^ (population density of 2394.3 inhabitants/km^2^), with males (50.3%) slightly outnumbering females. Following the Ministry of Health (MoH) policy for the control of the HIV/AIDS epidemic in Ethiopia, JUSH has implemented the HIV/AIDS prevention and control programme since 2002, providing the administration of ART regimens in a separate unit as the main component of the programme. ART regimens in Ethiopia consist of a generic low-cost fixed-dose combination (FDC) of two nucleoside reverse transcriptase inhibitors (NRTIs) and one non-nucleoside reverse transcriptase inhibitor (NNRTI), with first line regimens of lamivudine (3TC) combined with stavudine (d4T) or zidovudine (AZT), and either nevirapine (NVP) or efavirenz (EFV) [16]. The ART regimens in this study included the following: 1a (3TC+D4T+NVP), 1b (3TC+D4T+FFV), 1c (3TC+AZT+NVP), and 1d (3TC+AZT+EFV).

### 2.2. Study Population

All medical cards of HIV/AIDS patients aged ≥ 18 years who initiated ART treatment between January 2006 and December 2010 at JUSH were revised, and available data of potential predictors of survival were collected by trained nurses. Each patient had a chart/record with a distinctive identification number, known as the ART unique identification number.

### 2.3. Standard Follow-Up of HIV Patients on ART

According to the national HIV testing and counselling guideline, HIV patients should be followed-up routinely [17]. Once the HIV diagnosis was confirmed using rapid HIV antibody tests (KHB/STAT/PAK®/Unigold™ tiebreaker algorithm) at JUSH, patients were clinically examined, and laboratory tests such as the CD4 count, total WBC count, haemoglobin measurement, transaminases ALT/AST ratio, and TB screening were performed. During the study period, the viral load measurement was not available at JUSH. Thus, ART onset was primarily based on the HIV disease stage and on the degree of immune damage (CD4 counts). The following criteria were considered to initiate ART at JUSH: WHO Stage 4 disease irrespective of the CD4 cell count, a WHO Stage 3 disease with a CD4 cell count < 350/mm^3^, and a WHO Stage 1 or 2 disease with a CD4 cell count < 200/mm^3^. After ART onset, patients were evaluated within the next two weeks, and then every one or two months thereafter, during scheduled medical appointments at JUSH. The evaluation included the assessment of drug side effects, the disease progression, and clinical improvements/deterioration, including the identification of opportunistic infections (such as Pneumonia and TB) or recurrent problems. According to the programme guidelines, when a patient does not turn up for scheduled appointments and/or does not pick up the ART medicines, he/she is contacted by telephone and/or visited at home by health workers.

### 2.4. Variables

The outcome variables were the survival time in months and the HIV/AIDS-related events (i.e., dead or censored). The survival time was calculated in months, taking into account the dates of onset of ART and the occurrence of the event (death) or censoring. The censoring time was measured for individuals who were on ART until December 2010 or failed to follow-up. The evaluated risk factors were gender (male or female), baseline age, baseline CD4 count, baseline weight in Kg, baseline TB status (negative or positive), baseline opportunistic infection (no or yes), ART regimen (1a, 1b, 1c or 1d), and level of adherence to the ART regimen (low or high). Death was defined as confirmed HIV/AIDS-related death with the certification of death by a medical practitioner, or a verbal or telephone confirmation of death from a relative or friend. High adherence was defined as a 95% adherence based on pill counts at clinic visits, and poor or low adherence was defined as the failure to achieve this criterion [18].

### 2.5. Statistical Analysis

The mortality rate in this study is expressed as the number of deaths per 100 people per year at risk (PYAR). While the numerator of the mortality rate is the number of deaths identified during the follow-up period, the denominator is the sum of the total years that each person was followed-up. Kaplan-Meier survival curves were used to estimate the probability of death during the study period. The Log-rank test was used to compare the estimated survival curves according to gender. Semi-parametric (Cox regression) and parametric (exponential, Weibull, and log-logistic) [13] survival models were first applied to identify the best-fitting model for the time-to-HIV/AIDS death data, and then to calculate the hazard ratios (HRs) of the incidence of death.

The hazard function at time t for a particular patient with a set of p covariates (x_1_, x_2_, … x_p_) is given as follows [19]:

(1) $$h\left(t|X\right)=\;h_{0}\left(t\right)\exp\left(\beta_{1}x_{1}+\beta_{2}x_{2}+\ldots +\beta_{p}x_{p}\right)=h_{0}\left(t\right)\exp\left(\beta^{\prime }X\right)$$

where $\beta_{j}$ is the estimated parameter for the j^th^ covariate, $h_{0}\left(t\right)$ is the baseline hazard function, and **x** is the vector of the covariates. The baseline hazard function was assumed to follow a specific distribution when a fully parametric proportional hazard model was fitted to the data, whereas the semi-parametric (Cox proportional hazard) model had no such constraint.

The graphical evaluation method was used for the appropriateness of the Weibull model. The $\log\left\{-log\hat{S}\left(t\right)\right\}$ versus log(t) line is a straight line when the Weibull distribution is appropriate or reasonable. The exponential regression model is a special case of the Weibull model with a shape parameter equal to 1, which leads to a constant hazard function. For the exponential model, the $\log\hat{S}\left(t\right)$ versus time plot should yield a straight line [19]. For the log-logistic model, a plot of log(1−$\hat{S}\left(t\right)$)/($\hat{S}\left(t\right)$) versus log(t) with a positive slope “p”, or log($\hat{S}\left(t\right)$)/(1−$\hat{S}\left(t\right)$) versus log(t) with a negative slope ‘p’, should be linear [19,20]. The relevant functions for the different parametric models for this study data set are plotted in Supplementary file Figure S1.

The Akaike information criterion (AIC) was used to compare the three parametric models. The model with the lowest AIC value was considered to be the best model for fitting the data. The AIC was calculated using the following formula [19]:

(2) AIC=−2*log(likelihood)+2(p+1+s)

where *p* denotes the number of covariates in the model, not including the constant terms; s = 0 stands for the exponential model; and s = 1 represents the Weibull and log-logistic models.

The goodness-of-fit of the semi-parametric and parametric survival models was tested by plotting the cumulative hazard rate against the Cox-Snell residuals of each model. The cumulative hazard rates of the models that fell closer to the referent line were considered to indicate the models with a better adherence to their assumptions. The Cox-Snell residual for the i^th^ individual at observed time t_i_ was defined as [19]:

(3) $$r_{ci}=\hat{H}\left(t_{i}|x_{i}\right)=-log\left[\hat{S}(t_{i}|x_{i})\right]$$

where t_i_ is the observed survival time for individual i, x_i_ is the vector of covariate values for individual i, and $\hat{S}\left(t_{i}\right)$ is the estimated survival function on the fitted value.

### 2.6. Ethical Approval

Ethical approval for the data collection of this study was obtained from the Jimma University Health Science Research Office (Reference number: RPGS/520/2011). The trained nurses collected the data with the administered questionnaire from standard medical registration cards of patients in the record office at the ART unit.

## 3. Results

The majority of the 456 adult HIV/AIDS patients included in the study who attended JUSH between 2006 and 2010 were female (312, 68.4%) (Table 1), and had initiated ART treatment before the age of 35 (median: 30 years, inter-quartile range (IQR: 23–37 years). The median baseline body weight and CD4 count of the patients were 51.0 kg (IQR: 45.0–57.0 kg) and 151.5 cells/µL (IQR: 90.75–217.2 cells/µL), respectively. The follow-up until December 2010 accounted for 1245 person-years at risk (PYAR) (857 and 388 PYAR for females and males respectively), and resulted in 66 (14.5%) deaths (41 females and 25 males) and 390 censored individuals, representing a median survival time of 34.0 months (IQR: 22.8–42.0 months). The overall mortality rate was 5.3/100 PYAR, with rates for males (6.5/100 PYAR) being higher than for females (4.8/100 PYAR). Forty deaths (60.6%) occurred early in the first year after the onset of ART. Most deaths were in patients that, at the time of the treatment onset, were older than 35 years (45.5%), had a TB infection confirmed (59.1%), and showed WHO clinical III or IV stages (53.1%). Deaths were more likely to occur among individuals who maintained a low adherence to the ART regimen than among those who maintained high adherence (*p* < 0.001). Deaths during the follow-up period were also more frequent among individuals who began ART in the late clinical stage IV (*p* < 0.001), compared to those who began ART in WHO stage I.

**Figure 1:**
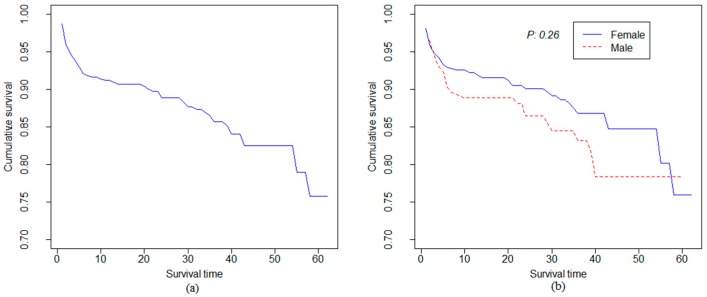
(**a**) Kaplan-Meier survival function curve for all individuals; (**b**) Kaplan-Meier survival function curve by gender with Log-rank test *p*-value.

According to the Kaplan Meier curves, the survival of the HIV-patients decreased quickly during the first seven months and then tailed off gradually, to reach its minimum value (75% survival) at the end of the follow-up (month 60^th^; Figure 1A). Survival was higher in females than in males during nearly the entire study period (except in the final months), but this difference was not significant (*p* = 0.26) (Figure 1B). The estimated survivorship functions do not reach zero, which indicates that the greatest observed survival time in the study was a censored value.

**Figure 2:**
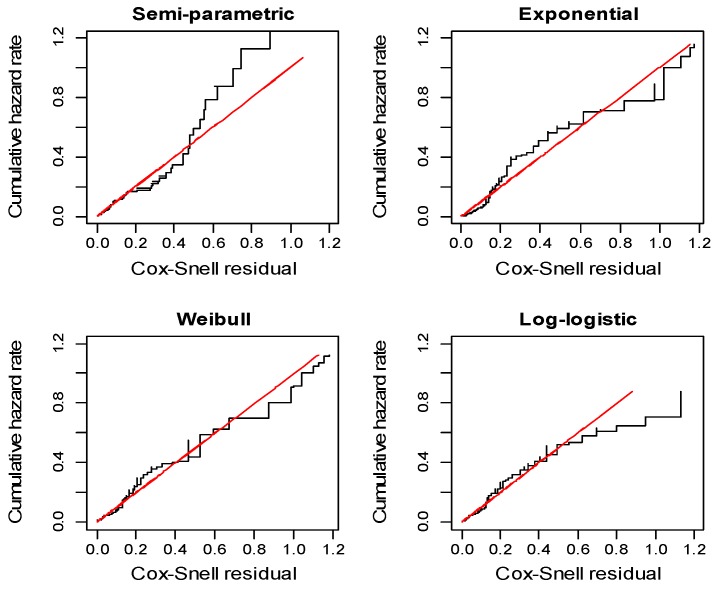
Cox-Snell residuals plot (Black line is the cumulative hazard, and Red line is the reference line with slope=1.0 and intercept=0) to evaluate the model fits of the semi-parametric and parametric survival models.

The Weibull regression model was the best model for fitting the data among the parametric models, since it has the lowest AIC value (492). Additionally, this was the model with the best adherence to the model assumptions (cumulative hazard closer to the reference line), as illustrated in the Cox-Snell residuals plots (Figure 2).

**Figure 3:**
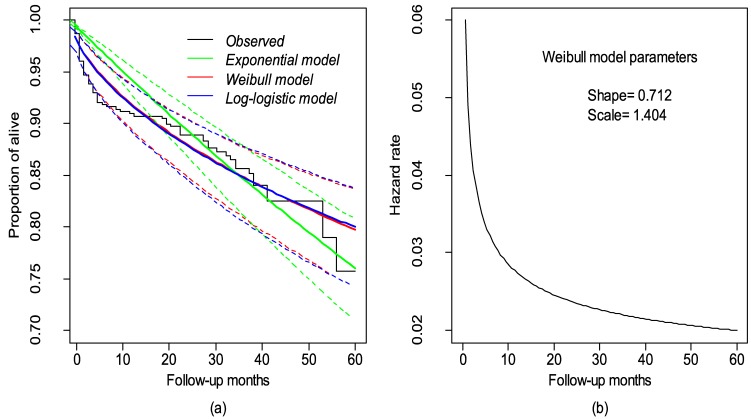
(**a**) The observed and estimated proportion of alieved individuals based on parametric survival models; (**b**) The hazard rate of the selected Weibull survival model with the shape and scale parameters.

Figure 3A indicates that the Weibull survival estimates are also in good agreement with the observed survival estimates, while Figure 3B indicates the evolution of a death rate pattern with time. A high risk of death occurred at the beginning of the ART regimen, and this risk decreased with time (decreasing hazard rate; shape parameter < 1).

In the multivariate Weibull regression model, the age of the patient at the beginning of the treatment, baseline body weight, baseline disease stage, and adherence to the ART regimen during the follow-up, were significantly associated with the time to HIV/AIDS-related mortality (Table 2). The patients who began ART at ages above 35 years exhibited a significantly higher death hazard than those who began treatment at an age below 25 years (AHR = 3.8, 95% CI: 1.6–9.1), and the baseline weight in kilograms at the beginning of the treatment was inversely associated with the time to death (AHR = 0.93, 95% CI: 0.90–0.97). Additionally, the patients in the advanced stage IV of the disease at the beginning of ART exhibited an increased risk of dying compared to those who began in the early stage I (AHR = 6.2, 95% CI: 2.2–14.2). Moreover, the individuals with low adherence to the ART regimen were more likely to present with fatal events than those with high adherence (AHR = 4.1, 95% CI: 2.5–7.1). Gender, baseline CD4 count, history of TB co-infection, history of opportunistic infections, and type of ART regimen, were not statistically significantly associated with the time until HIV/AIDS-related death.

## 4. Discussion

The Weibull survival regression model allowed for a more accurate identification of the risk factors for HIV-related mortality incidence rates in a non-concurrent retrospective cohort of patients with HIV/AIDS, who initiated ART regimen and were followed at Jimma University Specialized Hospital in Ethiopia between January 2006 and December 2010. The death rates decreased with time and were associated with an increased age and an advanced stage of the disease at the beginning of the treatment, as well as with poor adherence to the ART regimen during the study period.

### 4.1. HIV/AIDS Mortality Rate

Mortality in the first year of follow-up in our study (60%) was consistent with findings of a study in South Omo, Ethiopia, where 62.9% of patients died in the first year after the onset of ART [11]. Other retrospective cohort studies in Northern and North-western parts of Ethiopia have also reported similar death figures, with between 56% and 59% of total individuals dying before completing the first year of treatment [21,22]. High mortality rates during the first months after beginning the ART regimen were also reported through prospective cohort studies in Tanzania and South Africa [4,23], and were strongly associated with anaemia, thrombocytopenia, and severe malnutrition [4,23,24]. In Ethiopia, it was further found that early high death rates mainly occurred in patients with an advanced disease stage [25], as also reported in studies conducted in sub-Saharan Africa countries [26].

The mortality rates in our study (5.3/100 PYAR) are slightly lower than those reported in countries like Uganda and South Korea [27,28], but higher than those reported in other regions of Ethiopia (e.g., the Arbaminch city, the Amhara region, and the capital city Addis Ababa) [22,27,29,30]. For instance, the mortality rate estimated at Arbaminch hospital was 9.1 per 100 PYAR [27], and was 3.4 per 100 PYAR at University of Gonder Hospital, in North-western Ethiopia [22].

### 4.2. Survival Models

Comparisons of survival models under different distributions of the hazard function provide the best model for fitting the specific data with appropriate inference [31]. In our study, the Weibull survival model exhibited the smallest AIC, indicating its ability to fit the data. Previous longitudinal studies in Australia and England have also recognized the Weibull regression model as the best model for fitting the time until HIV/AIDS-related death data [3,32]. Our findings also agree with a study that compared parametric models for breast cancer survival data in India [14].

One of the weaknesses of the semi-parametric Cox model is that it makes the analyst focus on the regression coefficients, without considering the underlying distribution [15]. However, investigators have developed parametric survival models that lead to more precise estimations of survival probabilities and a better understanding of the event evolution during the time of study [3,14,15,28,33]. Hence, parametric models may be superior to semiparametric models in this setting because they allow for explicit modelling of the underlying death risk (baseline hazard) [15].

### 4.3. Factors Associated with the Risk of Death

The significance of gender in determining the survival time until death is variable in many studies [7,24,25]. Although our study did not find any association between gender and the survival time until HIV/AIDS-related death, other studies in Ethiopia and abroad have reported that the mortality rate seems to be higher in males than in females [7,26]. Among possible reasons for the gender difference, it has been suggested that female patients tend to know about their HIV status at an earlier stage and begin antiretroviral therapy with better CD4 cell counts relative to males [7].

In contrast to other studies conducted in the Northern and Somali region of Ethiopia [10,22,29], our study did not demonstrate an association between baseline TB infection and death hazard rate, possibly because all of the HIV-TB co-infected patients received opportune TB treatment, in concordance with the DOTs treatment guidelines. A study in Northern Ethiopia showed that HIV/AIDS patients who developed TB had shorter survival times than TB-negative patients [29]; and in North-western Ethiopia, a study showed that the presence of a tuberculosis co-infection at ART onset was significantly associated with HIV/AIDS-related mortality [22] (Hazard ratio = 2.91; 95% CI: 2.11–4.02).

Our study found that an increased age (>35 years old) was associated with an increased death hazard rate. These results are consistent with findings from a study in China showing a strong association of the age at the beginning of ART treatment with HIV/AIDS-related death [24], but are in conflict with a study conducted in Addis Ababa, the capital of Ethiopia [9], and to other studies revealing no relationship of the age at the onset of ART treatment with the mortality rate [10,21,34]. Moreover, our findings showed that individuals who began the ART regimen with a low baseline weight and who were in the late clinical stage IV exhibited a greater risk of death. Those results support the recommendations to start ART at earlier stages, as previously stated in the Ethiopian Guidelines for the HIV counselling and testing (i.e., in all symptomatic persons at WHO stage IV, irrespective of CD4 cell counts [17]). Previous studies in Ethiopia, Tanzania, and China have also revealed that an advanced clinical stage at the initiation of ART is a significant predictor of mortality in HIV/AIDS patients under ART [3,7,9,24,29]. A study in Tanzania reported that patients who started ART in the advanced disease stage (WHO clinical stage IV) were four times more at risk of dying than early clinical stage patients [7]. Likewise, several studies showed that low body weight at the initiation of ART was significantly associated with HIV/AIDS-related mortality [7,10,21]. People who started ART with weights below 40 kg were dying at a 2.37 times higher rate than people with weights above 60 kg [21]. A study composed of two hospitals in “Shashemene” and “Assela”, in Ethiopia, however, found that the baseline weight was not a significant predicator of mortality [35]. Conversely, the WHO clinical stage at the beginning of ART was a significant predictor of mortality in this study.

The adherence level of people who undergo ART treatment was also significantly associated with HIV/AIDS-related death in this study. Patients with low ART treatment adherence had a four- times higher risk of dying compared to those with high adherence. Similar findings were found in studies conducted in the Somali region and the Northern Province of Cameron, reporting that low adherence to ART treatment is a significant predictor of mortality [10,36]. Successful antiretroviral therapy depends on sustaining high rates of adherence (i.e., correct dosage, taken on time, and in the correct manner, i.e., either with or without food). Educating a community regarding HIV testing services is essential for obtaining an earlier diagnosis, earlier initiation of ART, successful enrolment in ART treatment services, and efficient adherence counselling [27]. Complementary studies including quantitative and qualitative methodologies would help to better identify the determinants of the adherence of HIV patients to the ART, as well as the factors that influence the compliance of HIV case-management guidelines by health workers.

Our findings should be interpreted considering the nature of the study design. As a non-concurrent retrospective cohort, the study required data on exposure status at a specific earlier time-point (i.e., potential risk factors at the time of treatment onset), but also the identification of outcome events (i.e., death or censored) during the study period. Information on two important potential risk factors was not available in the healthcare records of HIV patients, i.e., JUSH: body mass index (BMI) [8,36,37,38] and viral load [39,40]. While the BMI could not be calculated due to the lack of the registered heights of patients in clinical records, measurements of viral loads were not available at the hospital during the study period. On the other hand, the outcome assessment of HIV patients and time at risk are highly dependent on the accomplishment of scheduled appointments by patients, as well as on the follow-up efforts and their registration on medical records by health workers at JUSH. Follow-up efforts were not quantified in our study, however, health workers at JUSH always attempt to follow the standard guidelines for the case-management of HIV patients, and those guidelines promote an appropriate follow-up.

## 5. Conclusions

Although study findings should be interpreted in consideration of the study design (i.e., retrospective collection of cohort data at a specialized hospital), our findings revealed high mortality rates in the earlier months after ART onset. The Weibull model was found to be the best fitting parametric model for the HIV/AIDS-related mortality data, allowing for the identification of the following factors associated with the mortality rate: age group older than 35 years, low baseline weight and advanced clinical stage IV at the beginning of ART, and low adherence to ART. The timely onset of ART treatment, and the promotion and monitoring of the adherence to ART treatment, should be important components of HIV/AIDS programmes.

## Figures and Tables

**Table 1:** Summary of HIV/AIDS-related mortality by different baseline characteristics of adult HIV patients included in the study, Jimma University Specialized Hospital, Southwest Ethiopia.

Covariates		Total	Death N (%)	Censored N (%)
Gender	Female	312	41 (13.1)	271 (86.9)
	Male	144	25 (17.4)	119 (82.6)
Age	<25	99	7 (7.1)	92 (92.9)
	25–35	224	29 (12.9)	195 (87.1)
	>35	133	30 (22.6)	103 (77.4)
CD4	≤200	316	44 (13.9)	272 (86.1)
	>200	140	22 (15.7)	118 (84.3)
TB status	Negative	247	27 (10.9)	220 (89.1)
	Positive	209	39 (18.7)	170 (81.3)
POI	No	255	26 (10.2)	229 (89.8)
	Yes	401	40 (19.9)	161 (80.1)
WHO stage	I	65	5 (7.7)	60 (92.3)
	II	149	20 (13.4)	129 (86.6)
	III	188	22 (11.7)	166 (88.3)
	IV	54	19 (35.2)	35 (64.8)
Adherence	Low	102	27 (26.5)	75 (73.5)
	High	354	39 (11.0)	315 (89.0)
Regimen	1a	366	49 (13.4)	317 (86.6)
	1b	48	9 (18.8)	39 (81.3)
	1c	30	6 (20.0)	24 (80.0)
	1d	12	2 (16.7)	10 (83.3)

**Table 2:** Multivariate analysis of factors associated to HIV/AIDS mortality in southwestern Ethiopia: Parameter estimate with the standard error, acceleration factor, and 95% confidence interval using the four potential survival models.

	Cox	Exponential	Weibull	Log-logistic
	AHR (95% C.I.)	AHR (95% C.I.)	AHR (95% C.I.)	ϕ (95% C.I.)
Gender				
Female	1	1	1	1
Male	1.42 (0.81–2.49)	1.59 (0.90–2.80)	1.46 (0.84–2.58)	0.63 (0.27–1.41)
Age				
<25	1	1	1	
25–35	1.56 (0.66–3.62)	1.53 (0.65–3.59)	1.56 (0.67–3.64)	0.53 (0.16–1.73)
≥35	3.45 * (1.45–8.24)	4.07 * (1.70–9.77)	3.81 * (1.60–9.08)	0.16 * (0.05–0.58)
BW	0.94 * (0.91–0.97)	0.93 * (0.89–0.95)	0.93 * (0.90–0.97)	1.08 * (1.04–1.14)
CD4				
≥200	1	1	1	1
<200	1.58 (0.89–2.79)	1.88 * (1.05–3.37)	1.73 (0.98–3.07)	0.53 (0.24–1.21)
TB				
Negative	1	1	1	1
Positive	1.28 (0.74–2.15)	1.32 (0.76–2.28)	1.33 (0.77–2.29)	0.67 (0.31–1.48)
OI				
No	1	1	1	1
Yes	1.38 (0.78–2.43)	1.46 (0.84–2.55)	1.44 (0.83–2.52)	0.59 (0.26–1.33)
Regimen				
1a	1	1	1	1
1b	0.78 (0.36–1.68)	0.65 (0.29–1.40)	0.73 (0.34–1.58)	1.25 (0.39–4.05)
1c	0.95 (0.38–2.32)	1.00 (0.41–2.46)	0.99 (0.41–2.44)	0.85 (0.22–3.25)
1d	1.81 (0.42–3.73)	2.20 (0.50–3.93)	2.04 (0.49–8.81)	0.39 (0.11–3.25)
WHO stage				
I	1	1	1	1
II	1.75 (0.64–4.79)	1.68 (0.62–4.57)	1.67 (0.62–4.52)	0.44 (0.11–1.84)
III	1.70 (0.63–4.58)	1.69 (0.63–4.54)	1.61 (0.61–4.32)	0.51 (0.02–2.03)
IV	5.68 (2.06–15.71)	7.1 * (2.56–11.93)	6.2 * (2.24–14.17)	0.07 * (0.06–0.35)
Adherence				
High	1	1	1	1
Low	3.78 * (2.23–6.42)	5.03 (2.96–8.55)	4.16 * (2.45–7.07)	0.14 * (0.06–0.33)
AIC	696	504	492	497

AHR: Adjusted Hazard Ratio; ϕ**:** Acceleration factor, it relates the factor with the survival time in log-logistic survival model; Significance at *p*-value < 0.05.

## Supplementary Materials

The following are available online at www.mdpi.com/1660-4601/14/3/296/s1, Figure S1: Different parametric models for data set in this study.
