# Supplementary material for: Risk Factors for Mortality among Adult HIV/AIDS Patients Following Antiretroviral Therapy in Southwestern Ethiopia: An Assessment through Survival Models
Source: Int J Environ Res Public Health. 2017 Mar 12;14(3):296. doi: 10.3390/ijerph14030296 (PMC5369132; doi:10.3390/ijerph14030296)
Supplement: Supplementary file 1 [file ijerph-14-00296-s001.pdf]

Supplementary Materials: Risk Factors for Mortality among Adult HIV/AIDS Patients Following Antiretroviral Therapy in Southwestern Ethiopia: An Assessment through Survival Models

Dinberu Seyoum, Jean-Marie Degryse,Yehenew Getachew Kifle, Ayele Taye, Mulualem Tadesse, Belay Birlie, Akalu Banbeta, Angel Rosas-Aguirre, Luc Duchateau and Niko Speybroeck

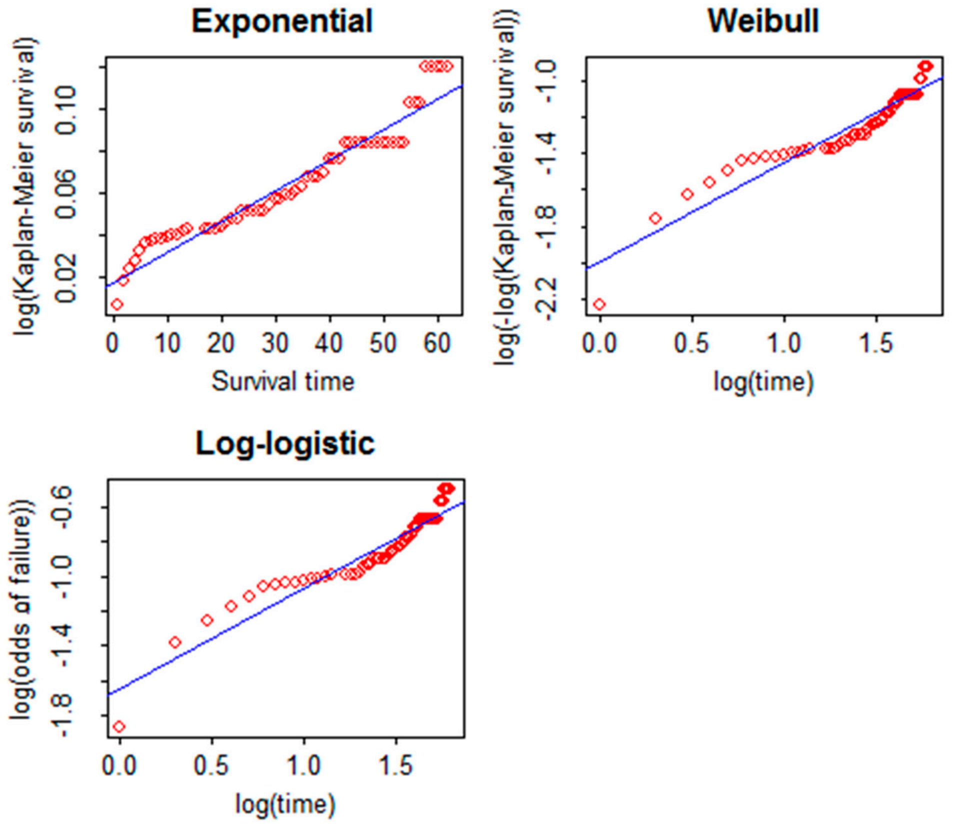

Figure S1. Different parametric models for data set in this study.

© 2017 by the authors; licensee MDPI, Basel, Switzerland. This article is an open access article distributed under the terms and conditions of the Creative Commons by Attribution (CC-BY) license (http://creativecommons.org/licenses/by/4.0/).
